# Supplementary material for: Artificial intelligence-enabled prediction of chemotherapy-induced cardiotoxicity from baseline electrocardiograms
Source: Nat Commun. 2024 Mar 21;15:2536. doi: 10.1038/s41467-024-45733-x (PMC10957877; doi:10.1038/s41467-024-45733-x)
Supplement: Supplementary file 1 — Supplementary Information [file 41467_2024_45733_MOESM1_ESM.pdf]

Supplementary Materials

Supplementary Tables

Supplementary Table 1 Baseline demographics and clinical characteristics of the enrolled patients treated with anthracyclines, stratified by the occurrence of CTRCD in BWH

		CTRCD		
	**All (n=637)**	**No (n=582)**	**Yes (n=55)**	**P-value**
Age, years (SD)	55.4 (16.3)	55.4 (16.2)	55.3 (17.1)	0.957
Male, n (%)	296 (46.5)	265 (45.5)	31 (56.4)	0.162
Race, n (%)				0.194
White	543 (85.2)	500 (85.9)	43 (78.2)	
Black	28 (4.4)	26 (4.5)	2 (3.6)	
Asian	19 (3.0)	17 (2.9)	2 (3.6)	
Other	47 (7.4)	39 (6.7)	8 (14.5)	
Diagnosis, n (%)				<0.001
Lymphoma	209 (32.8)	194 (33.3)	15 (27.3)	
Leukemia	236 (37.0)	202 (34.7)	34 (61.8)	
Breast cancer	92 (14.4)	91 (15.6)	1 (1.8)	
Sarcoma	47 (7.4)	43 (7.4)	4 (7.3)	
Other	53 (8.3)	52 (8.9)	1 (1.8)	
LVEF at baseline, % (SD)	62.9 (5.6)	63.0 (5.6)	61.0 (4.7)	0.011
Comorbidities, n (%)				
CAD	19 (3.0)	15 (2.6)	4 (7.3)	0.123
Hypertension	157 (24.6)	146 (25.1)	11 (20.0)	0.501
Diabetes	61 (9.6)	57 (9.8)	4 (7.3)	0.713
Dyslipidemia	190 (29.8)	171 (29.4)	19 (34.5)	0.518
Obesity	207 (32.5)	186 (32.0)	21 (38.2)	0.429
Smoke, n (%)	294 (46.2)	267 (45.9)	27 (49.1)	0.752
Initial anthracycline dose, mg/m^2, median [IQR]	40.0 [25.0,60.0]	40.0 [25.0,60.0]	50.0 [30.0,60.0]	0.17
Cumulative anthracycline dose, mg/m^2, median [IQR]	200 [101, 298]	200 [100, 298]	206 [169, 299]	0.16
ECG abnormalities, n (%)				
1AVb	11 (1.7)	100 (17.2)	23 (41.8)	<0.001
Atrial fibrillation	14 (2.2)	9 (1.5)	2 (3.6)	0.551
RBBB	8 (1.3)	12 (2.1)	2 (3.6)	0.779
LBBB	7 (1.1)	6 (1.0)	2 (3.6)	0.305
ST-T change	7 (1.1)	7 (1.2)	0 (0.0)	0.888
LVH	58 (9.1)	6 (1.0)	1 (1.8)	1
PAC	33 (5.2)	55 (9.5)	3 (5.5)	0.46
PVC	9 (1.4)	31 (5.3)	2 (3.6)	0.824
Low voltage	7 (1.1)	8 (1.4)	1 (1.8)	1
Paced rhythm	7 (1.1)	7 (1.2)	0 (0.0)	0.888
Any abnormality	141 (22.1)	6 (1.0)	1 (1.8)	1

A $\chi 2$ test (two-sided) was used to identify differences in proportions across race and diagnosis. A Wilcoxon test (two-sided) was used for initial anthracycline dose and cumulative anthracycline dose, and a t-test (two-sided) was used for other variables. No adjustments were made for multiple comparisons. CTRCD: cancer therapy-related cardiac dysfunction, LVEF: left ventricular ejection fraction, CAD: coronary artery disease, ECG: electrocardiogram, 1AVb: first degree atrio-ventricular block, RBBB: right bundle branch block, LBBB: left bundle branch block, LVH: left ventricular hypertrophy, PAC: premature atrial contraction, PVC: premature ventricular contraction, SD: standard deviation, IQR: interquartile range. N represents number of patients.

Supplementary Table 2 Baseline demographics and clinical characteristics of the enrolled patients treated with anthracyclines, stratified by the occurrence of CTRCD in MGH

		CTRCD		
	**All (n=501)**	**No (n=457)**	**Yes (n=44)**	**P-value**
Age, mean (SD)	58.6 (16.3)	58.4 (16.3)	60.2 (16.2)	0.494
Male, n (%)	243 (48.5)	221 (48.4)	22 (50.0)	0.96
Race, n (%)				0.018
White	411 (82.0)	381 (83.4)	30 (68.2)	
Black	20 (4.0)	15 (3.3)	5 (11.4)	
Asian	23 (4.6)	19 (4.2)	4 (9.1)	
Other	47 (9.4)	42 (9.2)	5 (11.4)	
Diagnosis, n (%)				0.475
Lymphoma	230 (45.9)	211 (46.2)	19 (43.2)	
Leukemia	122 (24.4)	107 (23.4)	15 (34.1)	
Breast cancer	67 (13.4)	64 (14.0)	3 (6.8)	
Sarcoma	48 (9.6)	44 (9.6)	4 (9.1)	
Other	34 (6.8)	31 (6.8)	3 (6.8)	
LVEF at baseline, % (SD)	67.5 (6.4)	67.9 (6.2)	63.9 (7.6)	<0.001
Comorbidities, n (%)				
CAD	37 (7.4)	31 (6.8)	6 (13.6)	0.174
Hypertension	171 (34.1)	151 (33.0)	20 (45.5)	0.136
Diabetes	55 (11.0)	48 (10.5)	7 (15.9)	0.399
Dyslipidemia	201 (40.1)	180 (39.4)	21 (47.7)	0.359
Obesity	169 (33.7)	153 (33.5)	16 (36.4)	0.826
Smoke, n (%)	236 (47.1)	215 (47.0)	21 (47.7)	1
Initial anthracycline dose, mg/m^2, median [IQR]	40.0 [30.0, 50.0]	40.0 [30.0, 50.0]	30.0 [30.0, 50.0]	0.51
Cumulative anthracycline dose, $mg/m^{2,}$ median [IQR]	150 [89, 246]	150 [89, 247]	150 [90, 239]	0.83
ECG abnormalities, n (%)				
1AVb	9 (1.8)	68 (14.9)	7 (15.9)	1
Atrial fibrillation	14 (2.8)	8 (1.8)	1 (2.3)	1
RBBB	15 (3.0)	11 (2.4)	3 (6.8)	0.224
LBBB	6 (1.2)	14 (3.1)	1 (2.3)	1
ST-T change	2 (0.4)	2 (0.4)	4 (9.1)	<0.001
LVH	38 (7.6)	2 (0.4)	0 (0.0)	1
PAC	35 (7.0)	36 (7.9)	2 (4.5)	0.618
PVC	10 (2.0)	31 (6.8)	4 (9.1)	0.792
Low voltage	3 (0.6)	8 (1.8)	2 (4.5)	0.483
Paced rhythm	4 (0.8)	3 (0.7)	0 (0.0)	1
Any abnormality	122 (24.4)	3 (0.7)	1 (2.3)	0.792

A χ2 test (two-sided) was used to identify differences in proportions across race and diagnosis. A Wilcoxon test (two-sided) was used for initial anthracycline dose and cumulative anthracycline dose, and a t-test (two-sided) was used for other variables. No adjustments were made for multiple comparisons. CTRCD: cancer therapy-related cardiac dysfunction, LVEF: left ventricular ejection fraction, CAD: coronary artery disease, ECG: electrocardiogram, 1AVb: first degree atrio-ventricular block, RBBB: right bundle branch block, LBBB: left bundle branch block, LVH: left ventricular hypertrophy, PAC: premature atrial contraction, PVC: premature ventricular contraction, SD: standard deviation, IQR: interquartile range N represents number of patients.

Supplementary Table 3 Baseline demographics and clinical characteristics of the enrolled patients treated with anthracyclines, stratified by the occurrence of CTRCD in Keio

		CTRCD		
	All (n=190)	No (n=183)	Yes (n=7)	P-value
Age, mean (SD)	61.0 (18.2)	61.2 (18.2)	55.1 (18.8)	0.389
Male, n (%)	95 (50.0)	93 (50.8)	2 (28.6)	0.441
Race, n (%)				
Asian	501 (100)	457 (100)	44 (100)	1
Diagnosis, n (%)				0.846
Lymphoma	28 (14.7)	27 (14.8)	1 (14.3)	
Leukemia	91 (47.9)	88 (48.1)	3 (42.9)	
Breast cancer	21 (11.1)	20 (10.9)	1 (14.3)	
Sarcoma	32 (16.8)	30 (16.4)	2 (28.6)	
Other	18 (9.5)	18 (9.8)	0 (0.0)	
LVEF at baseline, % (SD)	64.9 (8.2)	64.9 (8.3)	64.5 (8.0)	0.915
Comorbidities, n (%)				
CAD	22 (11.6)	22 (12.0)	0 (0.0)	0.709
Hypertension	50 (26.3)	48 (26.2)	2 (28.6)	1
Diabetes	33 (17.4)	31 (16.9)	2 (28.6)	0.773
Dyslipidemia	31 (16.3)	30 (16.4)	1 (14.3)	1
Obesity	19 (10.0)	18 (9.8)	1 (14.3)	1
Smoke, n (%)	72 (37.9)	68 (37.2)	4 (57.1)	0.501
Initial anthracycline dose, mg/m^2, median [IQR]	48.7 [28.7, 50.1]	48.3 [26.9, 50.1]	49.9 [39.5, 62.5]	0.40
ECG abnormalities, n (%)				
1AVb	9 (4.7)	8 (4.4)	1 (14.3)	0.76
Atrial fibrillation	7 (3.7)	7 (3.8)	0 (0.0)	1
RBBB	9 (4.7)	8 (4.4)	1 (14.3)	0.76
LBBB	4 (2.1)	4 (2.2)	0 (0.0)	1
ST-T change	12 (6.3)	11 (6.0)	1 (14.3)	0.927
LVH	10 (5.3)	10 (5.5)	0 (0.0)	1
PAC	9 (4.7)	9 (4.9)	0 (0.0)	1
PVC	2 (1.1)	2 (1.1)	0 (0.0)	1
Low voltage	3 (1.6)	3 (1.6)	0 (0.0)	1
Paced rhythm	1 (0.5)	1 (0.5)	0 (0.0)	1
Any abnormality	47 (24.7)	45 (24.6)	2 (28.6)	1

A $\chi 2$ test (two-sided) was used to identify differences in proportions across race and diagnosis. A Wilcoxon test (two-sided) was used for initial anthracycline dose, and a t-test (two-sided) was used for other variables. No adjustments were made for multiple comparisons. CTRCD: cancer therapy-related cardiac dysfunction, LVEF: left ventricular ejection fraction, CAD: coronary artery disease, ECG: electrocardiogram, 1AVb: first degree atrio-ventricular block, RBBB: right bundle branch block, LBBB: left bundle branch block, LVH: left ventricular hypertrophy, PAC: premature atrial contraction, PVC: premature ventricular contraction, SD: standard deviation, IQR: interquartile range. N represents number of patients.

Supplementary Table 4: Baseline demographics and clinical characteristics of the enrolled patients treated with anthracyclines, stratified by the occurrence of CTRCD in the training cohort

		CTRCD		
	All (n=317)	No (n=299)	Yes (n=18)	P-value
Age, mean (SD)	58.6 (17.6)	58.6 (17.7)	58.3 (17.4)	0.955
Male, n (%)	151 (47.6)	145 (48.5)	6 (33.3)	0.314
Race, n (%)				0.061
White	105 (33.1)	97 (32.4)	8 (44.4)	
Black	9 (2.8)	8 (2.7)	1 (5.6)	
Asian	194 (61.2)	187 (62.5)	7 (38.9)	
Other	9 (2.8)	7 (2.3)	2 (11.1)	
Diagnosis, n (%)				
Lymphoma	72 (22.7)	69 (23.1)	3 (16.7)	0.368
Leukemia	140 (44.2)	129 (43.1)	11 (61.1)	
Breast cancer	39 (12.3)	38 (12.7)	1 (5.6)	
Sarcoma	38 (12.0)	35 (11.7)	3 (16.7)	
Other	28 (8.8)	28 (9.4)	0 (0.0)	
LVEF at baseline, % (SD)	64.3 (7.2)	64.4 (7.3)	62.5 (5.8)	0.274
Comorbidities, n (%)				
CAD	26 (8.2)	25 (8.4)	1 (5.6)	1
Hypertension	84 (26.5)	79 (26.4)	5 (27.8)	1
Diabetes	46 (14.5)	43 (14.4)	3 (16.7)	1
Dyslipidemia	70 (22.1)	65 (21.7)	5 (27.8)	0.759
Obesity	127 (40.1)	37 (31.9)	6 (54.5)	0.237
Smoke, n (%)	43 (33.9)	116 (38.8)	11 (61.1)	0.103
Initial anthracycline dose, mg/m^2, median [IQR]	40.0 [30.0, 50.0]	40.0 [30.0, 50.0]	30.0 [30.0, 37.5]	0.21
ECG abnormalities, n (%)				
1AVb	9 (2.8)	8 (2.7)	1 (5.6)	1
Atrial fibrillation	10 (3.2)	9 (3.0)	1 (5.6)	1
RBBB	9 (2.8)	7 (2.3)	1 (5.6)	0.944
LBBB	8 (2.5)	8 (2.7)	1 (5.6)	1
ST-T change	23 (7.3)	12 (4.0)	1 (5.6)	1
LVH	13 (4.1)	21 (7.0)	2 (11.1)	0.856
PAC	20 (6.3)	20 (6.7)	0 (0.0)	0.526
PVC	12 (3.8)	12 (4.0)	0 (0.0)	0.818
Low voltage	3 (0.9)	3 (1.0)	0 (0.0)	1
Paced rhythm	3 (0.9)	3 (1.0)	0 (0.0)	1
Any abnormality	78 (24.6)	72 (24.1)	6 (33.3)	0.546

A $\chi 2$ test (two-sided) was used to identify differences in proportions across race and diagnosis. A Wilcoxon test (two-sided) was used for initial anthracycline dose, and a t-test (two-sided) was used for other variables. No adjustments were made for multiple comparisons. CTRCD: cancer therapy-related cardiac dysfunction, LVEF: left ventricular ejection fraction, CAD: coronary artery disease, ECG: electrocardiogram, 1AVb: first degree atrio-ventricular block, RBBB: right bundle branch block, LBBB: left bundle branch block, LVH: left ventricular hypertrophy, PAC: premature atrial contraction, PVC: premature ventricular contraction, SD: standard deviation, IQR: interquartile range. N represents number of patients.

Supplementary Table 5. Performance of the models using clinical variables on the deployment simulation cohort

	Description	AUROC (95%CI)	Sensitivity	PPV	N of pre-screened patients	N of patients detected	N of additional cases detected
Model 1	Full + AI	78.1 (72.2-84.0)	93.5	15.4	545	84	32
Model 2	4 var + AI	75.0 (69.2-80.8)	90.5	14.8	546	81	29
Model 3	AI only	67.2 (60.3-74.0)	71.9	12.5	519	65	13
Model 4	4 var w/o AI	67.2 (61.5-74.4)	80.9	13.5	541	73	21
Model 5	Full w/o AI	73.8 (67.6-80.1)	87.5	14.6	541	79	27
Random		50	52.0	9	572	52	

"Full model" includes age, sex, race, cancer type, initial anthracycline dose, and past medical histories, and "4 var model" includes age, sex, race, and cancer type. AI: AI-CTRCD score, AUROC: time-dependent area under the receiver operating curve for 2 years, CI: confidence interval, PPV: positive predictive value

Supplementary Table 6. Performance of AI-EF model detecting low left ventricular ejection fraction from electrocardiogram used in the study.

	BWH	MGH	UCSF	Keio
AUROC<br>(95%CI)	0.91<br>(0.89-0.93)	0.89<br>(0.86-0.91)	0.91<br>(0.88-0.93)	0.92<br>(0.90-0.93)

An AI model was trained after eliminating ECGs that were included in the CTRCD study population in the BWH cohort and tested in the same population (BWH, MGH, UCSF, Keio) in the previous study. AUROC: area under the receiver operating curve, BWH: Brigham and Women's Hospital, MGH: Massachusetts General Hospital, UCSF: University of California San Francisco, Keio: Keio University Hospital, CI: confidence interval

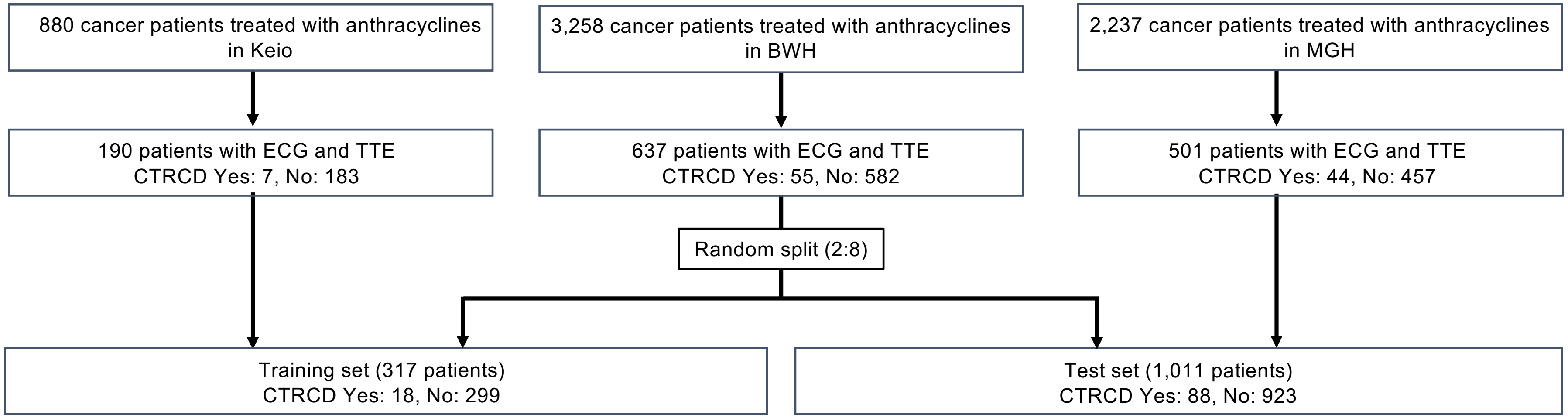

Supplementary Fig. 1: Selection of study population.

BWH: Brigham and Women's Hospital, MGH: Massachusetts General Hospital, ECG: electrocardiogram, TTE: transthoracic echocardiogram, CTRCD: cancer therapy-related cardiac dysfunction

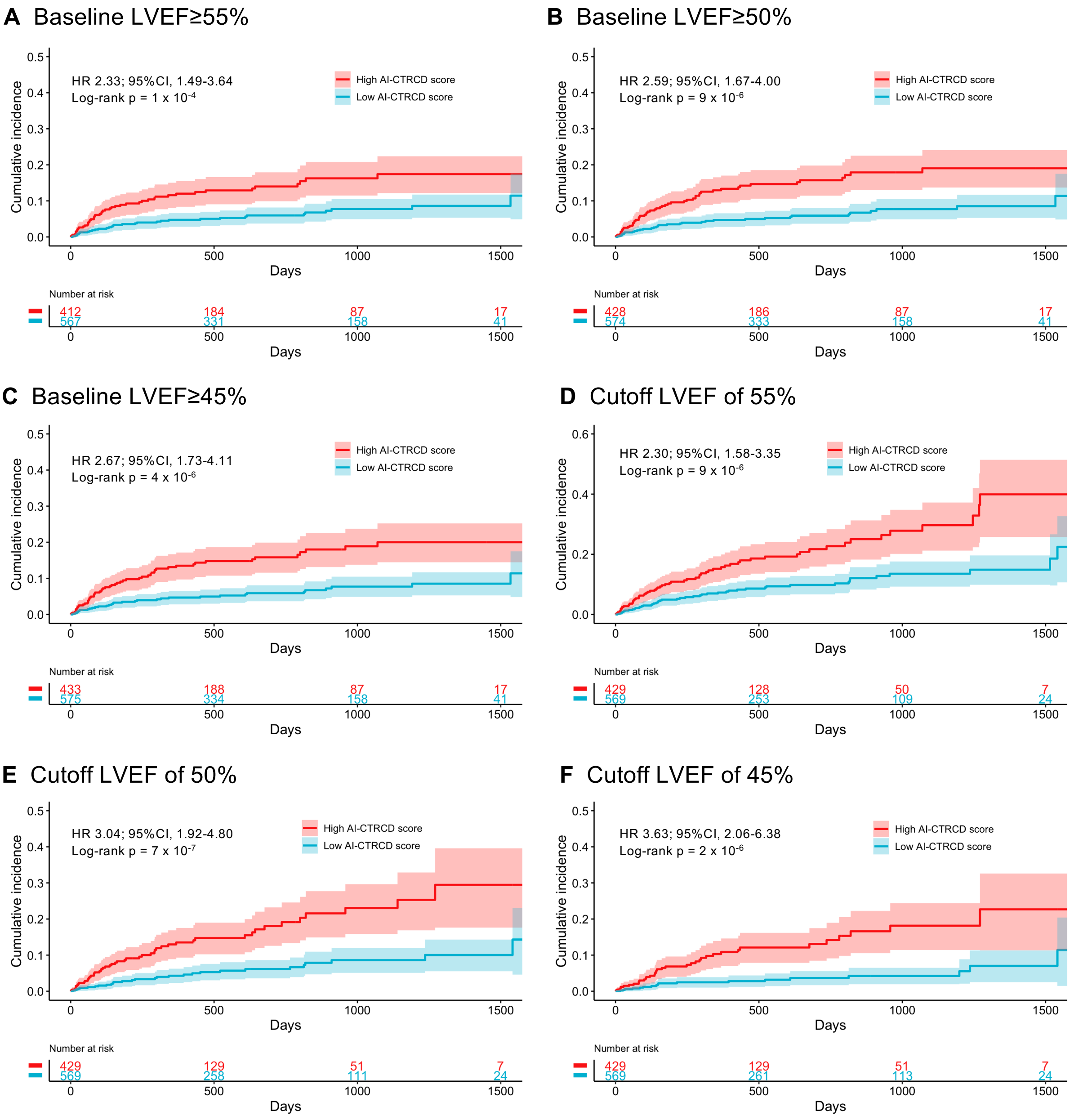

Supplementary Fig. 2: Sensitivity analysis regarding LVEF values.

Kaplan-Meyer plot showing the cumulative incidence of CTRCD between high and low AI-CTRCD scores in patients with a baseline LVEF of (**A**) ≥55%, (**B**) ≥50%, and (**C**) ≥45%, and using cutoff LVEF values of (**D**) 60%, (**E**) 50% and (**F**) 45% for the diagnosis of CTRCD instead of 53% in the main analysis. The transparent ribbons indicate 95%CI. Source data are provided as a Source Data file. CTRCD: cancer therapy-related cardiac dysfunction, AI: artificial intelligence, HR: hazard ratio, CI: confidence interval, LVEF: left ventricular ejection fraction.

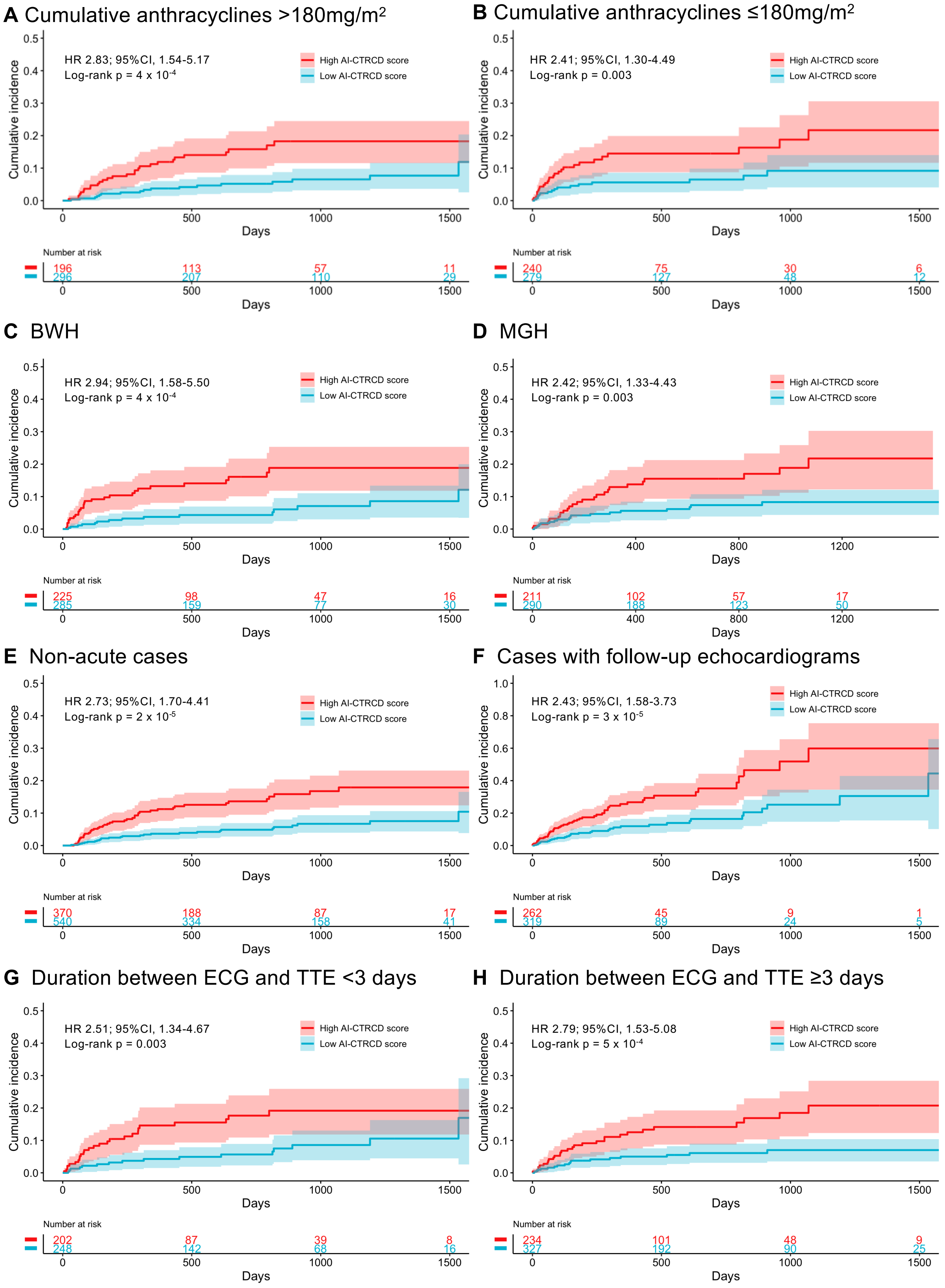

Supplementary Fig. 3: Sensitivity analyses regarding patient characteristics.

Kaplan-Meyer plot showing the cumulative incidence of CTRCD between high and low AI-CTRCD score in (**A**) patients who received >180mg/m^2 of cumulative anthracyclines (**B**) patients who received ≤180mg/m^2 of cumulative anthracyclines (**C**) patients recruited from BWH, (**D**) patients recruited from MGH, (**E**) cases experiencing events after 30 days from initiation of chemotherapy, (**F**) cases with at

least one follow-up echocardiogram, (**G**) cases with duration between ECG and TTE <3 days, and (**H**) cases with duration between ECG and TTE ≥3 days. The transparent ribbons indicate 95%CI. Source data are provided as a Source Data file. CTRCD: cancer therapy-related cardiac dysfunction, AI: artificial intelligence, HR: hazard ratio, CI: confidence interval, BWH: Brigham and Women's Hospital, MGH: Massachusetts General Hospital. ECG: electrocardiogram, TTE: transthoracic echocardiogram

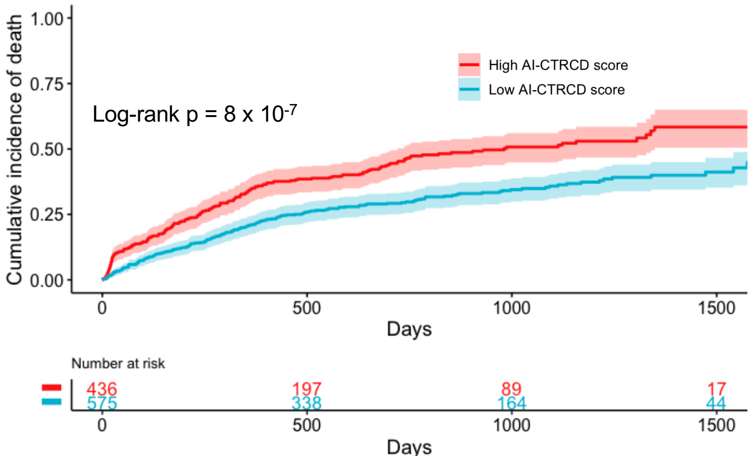

Supplementary Fig. 4: Cumulative incidence of death in patients with high and low AI-CTRCD scores.

Kaplan-Meier plot showing the cumulative incidence of death between high and low AI-CTRCD scores. Patients categorized in the high AI-CTRCD score group experienced more deaths compared to patients in the low AI-CTRCD score group. Source data are provided as a Source Data file. CTRCD: cancer therapy-related cardiac dysfunction, AI: artificial intelligence

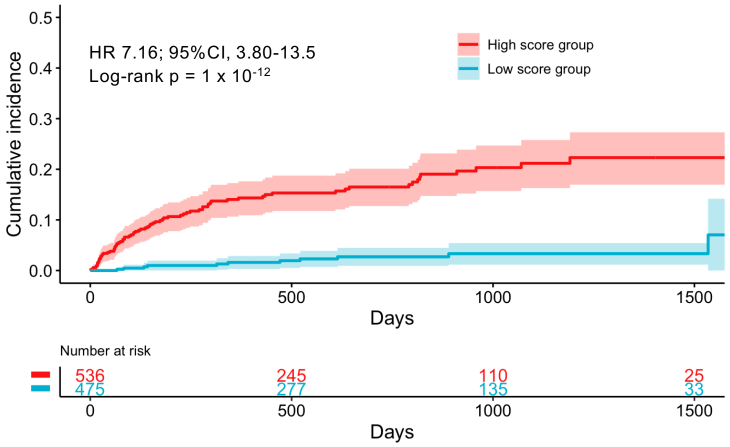

Supplementary Fig. 5: Cumulative incidence of CTRCD stratified by the model including AI-CTRCD score and clinical variables

Patients with high model scores were 7 times higher at risk of CTRCD compared to those with low model scores. Source data are provided as a Source Data file. CTRCD: cancer therapy-related cardiac dysfunction, AI: artificial intelligence, HR: hazard ratio, CI: confidence interval.
