# Supplementary material for: Artificial intelligence-enabled prediction of chemotherapy-induced cardiotoxicity from baseline electrocardiograms
Source: Nat Commun. 2024 Mar 21;15:2536. doi: 10.1038/s41467-024-45733-x (PMC10957877; doi:10.1038/s41467-024-45733-x)
Supplement: Supplementary file 3 — Reporting Summary [file 41467_2024_45733_MOESM3_ESM.pdf]

nature portfolio

Corresponding author(s): Rahul C. Deo

Last updated by author(s): Nov 21, 2023

Reporting Summary

Nature Portfolio wishes to improve the reproducibility of the work that we publish. This form provides structure for consistency and transparency in reporting. For further information on Nature Portfolio policies, see our Editorial Policies and the Editorial Policy Checklist.

Statistics

For all statistical analyses, confirm that the following items are present in the figure legend, table legend, main text, or Methods section.

n/a	Confirmed	
☐	☒	The exact sample size (n) for each experimental group/condition, given as a discrete number and unit of measurement
☐	☒	A statement on whether measurements were taken from distinct samples or whether the same sample was measured repeatedly
☐	☒	The statistical test(s) used AND whether they are one- or two-sided<br>*Only common tests should be described solely by name; describe more complex techniques in the Methods section.*
☐	☒	A description of all covariates tested
☒	☐	A description of any assumptions or corrections, such as tests of normality and adjustment for multiple comparisons
☐	☒	A full description of the statistical parameters including central tendency (e.g. means) or other basic estimates (e.g. regression coefficient) AND variation (e.g. standard deviation) or associated estimates of uncertainty (e.g. confidence intervals)
☐	☒	For null hypothesis testing, the test statistic (e.g. F, t, r) with confidence intervals, effect sizes, degrees of freedom and P value noted<br>*Give P values as exact values whenever suitable.*
☒	☐	For Bayesian analysis, information on the choice of priors and Markov chain Monte Carlo settings
☒	☐	For hierarchical and complex designs, identification of the appropriate level for tests and full reporting of outcomes
☒	☐	Estimates of effect sizes (e.g. Cohen's d, Pearson's r), indicating how they were calculated

Our web collection on statistics for biologists contains articles on many of the points above.

Software and code

Policy information about availability of computer code

Data collection	All data were collected using Python 3.7.3 and Numpy 1.19.2
Data analysis	The AI models were developed using Tensorflow 2.6 in Python 3.7.3. Kaplan-Meier curves were plotted using survival package (3.5.7) and ggplot2 package (3.4.4) in R 3.6.1. Cox proportional hazard analyses were also performed using survival package in R. Fine and Gray model was constructed using cmprsk package (2.2.11). Codes for running the model are available as supplementary materials attached to the manuscript. A web-based environment to run our model is available at http://onebraveideaml.org.

For manuscripts utilizing custom algorithms or software that are central to the research but not yet described in published literature, software must be made available to editors and reviewers. We strongly encourage code deposition in a community repository (e.g. GitHub). See the Nature Portfolio guidelines for submitting code & software for further information.

Data

Policy information about availability of data

All manuscripts must include a data availability statement. This statement should provide the following information, where applicable:

- Accession codes, unique identifiers, or web links for publicly available datasets
- A description of any restrictions on data availability
- For clinical datasets or third party data, please ensure that the statement adheres to our policy

The data that support the findings of this study are available in the article, in the Supplementary Information, and on request from the corresponding author R.C.D. upon approval of the data sharing committees of the respective institutions. The data are not publicly available due to the presence of information that could

compromise research participant privacy. Data use agreement will be required for data sharing. Requests will be responded within 3 months by the corresponding author. Source data are provided with this paper.

Research involving human participants, their data, or biological material

Policy information about studies with human participants or human data. See also policy information about sex, gender (identity/presentation), and sexual orientation and race, ethnicity and racism.

Reporting on sex and gender	Self-reported sex was used for the entire analysis. Sex-based stratification analysis was performed because cardiovascular outcomes are known to be different between sex. We found that our findings were affected by sex.
Reporting on race, ethnicity, or other socially relevant groupings	We used self-reported race/ethnicity for our analyses. Information on self-reported race/ethnicity was obtained from electronic health records. Obesity was defined as a body mass index ≥30.
Population characteristics	Patient demographics including age, sex, race, and clinical characteristic including body weight, LVEF at baseline, comorbidities (coronary artery disease, hypertension, diabetes, dyslipidemia), and prescription data were obtained from the database. The cancer diagnoses, treatment drugs, and the dose of drugs were manually confirmed by chart review. Missing variables in the dataset were also manually filled from the chart.
Recruitment	Patient demographics data and prescription information were retrospectively obtained from the Massachusetts General Brigham Enterprise Data Warehouse, which includes elec-tronic health records from >20 provider locations across a large integrated delivery network in Massachusetts. Patients older than 18 years old who were treated with chemotherapy including at least one dose of anthracyclines (doxorubicin, idarubicin, daunorubicin, and epirubicin) were first identified. Among these subjects, those who had both an ECG and an echocardiogram ≤90 days before the initiation of chemotherapy were included in the study.
Ethics oversight	This study complies with all ethical regulations and guidelines. This study protocol was approved by the institutional review board of Mass General Brigham (2019P002651) and Keio University School of Medicine (approval number: 20200030). The IRB of participating institutions provides a waiver for individual consent if the research meets the following criteria: the study involves no more than minimal risk to the subjects; the waiver or alteration will not adversely affect the rights and welfare of the subjects; the research could not practicably be carried out without the waiver or alteration; and whenever appropriate, the subjects will be provided with additional pertinent information after participation. The IRB approved the waiver of consent since the study met these criteria. This study had minimal patient risk: it collected data retrospectively, there was no direct contact with patients, and data were collected after medical care was completed. The only minimal risk was the breach of confidentiality during data abstraction from the electronic health record system. It was considered impractical to perform the study with consent given the large number of participants and the need for using historical data where the patients were no longer in the system. Waiver of consent was considered essential to recruit an unbiased and representative cohort of patients.

Note that full information on the approval of the study protocol must also be provided in the manuscript.

Field-specific reporting

Please select the one below that is the best fit for your research. If you are not sure, read the appropriate sections before making your selection.

- [x] Life sciences
- [ ] Behavioural & social sciences
- [ ] Ecological, evolutionary & environmental sciences

For a reference copy of the document with all sections, see nature.com/documents/nr-reporting-summary-flat.pdf

Life sciences study design

All studies must disclose on these points even when the disclosure is negative.

Sample size	The numbers of patients enrolled were determined based on availability. The number of samples required for neural-network training is usually determined empirically. Our results showing good performance and generalizability support that the numbers were sufficient.
Data exclusions	Patients without baseline electrocardiogram or echocardiogram within 90 days prior to initial treatment were excluded.
Replication	The study results were replicated successfully in 2 academic medical centers.
Randomization	The patients in BWH were randomly allocated into training/ test cohorts using the random number generator implemented in Python, numpy and tensorflow.
Blinding	None of blinding procedure was performed given the retrospective nature of the study design

Reporting for specific materials, systems and methods

We require information from authors about some types of materials, experimental systems and methods used in many studies. Here, indicate whether each material, system or method listed is relevant to your study. If you are not sure if a list item applies to your research, read the appropriate section before selecting a response.

Materials & experimental systems

n/a	Involved in the study
☒	☐ Antibodies
☒	☐ Eukaryotic cell lines
☒	☐ Palaeontology and archaeology
☒	☐ Animals and other organisms
☒	☐ Clinical data
☒	☐ Dual use research of concern
☒	☐ Plants

Methods

n/a	Involved in the study
☒	☐ ChIP-seq
☒	☐ Flow cytometry
☒	☐ MRI-based neuroimaging
